# Effective Diagnosis of Alzheimer’s Disease *via* Multimodal Fusion Analysis Framework

**DOI:** 10.3389/fgene.2019.00976

**Published:** 2019-10-10

**Authors:** Xia-an Bi, Ruipeng Cai, Yang Wang, Yingchao Liu

**Affiliations:** ^1^Hunan Provincial Key Laboratory of Intelligent Computing and Language Information Processing, Hunan Normal University, Changsha, China; ^2^College of Information Science and Engineering, Hunan Normal University, Changsha, China

**Keywords:** Alzheimer’s disease, multimodal fusion analysis framework, functional magnetic resonance imaging, gene, disease diagnosis

## Abstract

Alzheimer’s disease (AD) is a complex neurodegenerative disease involving a variety of pathogenic factors, and the etiology detection of this disease has been a major concern of researchers. Neuroimaging is a basic and important means to explore the problem. It is the main current scientific research direction for combining neuroimaging with other modal data to dig deep into the potential information of AD through the complementarities among multiple data points. Machine learning methods possess great potentiality and have reached some achievements in this research area. A few studies have proposed some solutions to the effects of multimodal data fusion, however, the overall analytical framework for data fusion and fusion result analysis has thus far been ignored. In this paper, we first put forward a novel multimodal data fusion method, and further present a new machine learning framework of data fusion, classification, feature selection, and disease-causing factor extraction. The real dataset of 37 AD patients and 35 normal controls (NC) with functional magnetic resonance imaging (fMRI) and genetic data was used to verify the effectiveness of the framework, which was more accurate in classification and optimal feature extraction than other methods. Furthermore, we revealed disease-causing brain regions and genes, such as the olfactory cortex, insula, posterior cingulate gyrus, lingual gyrus, CNTNAP2, LRP1B, FRMD4A, and DAB1. The results show that the machine learning framework could effectively perform multimodal data fusion analysis, providing new insights and perspectives for the diagnosis of Alzheimer’s disease.

## Introduction

Alzheimer’s disease (AD) is an irreversible neuropsychiatric disorder, which often occurs in elderly and manifests clinically as memory deterioration, aphasia, social difficulties, and other symptoms (Morello et al., 2017; Tavana et al., 2018; Bregman et al., 2019). The mortality rate of AD is high and is rising every year compared with other brain diseases (Association, 2017; Association, 2018). This disease affects approximately 36 million people throughout the world with the incidence anticipated to triple by 2050 (Neville et al., 2015). Moreover, the complications caused by AD also make the patient miserable (Association, 2015). In the later stages of the disease, the patient not only needs to carry the costs of expensive cost treatment, but can also not take care of themselves and are therefore completely dependent on caregivers, placing a heavy burden on their families and society (Association, 2016). Early diagnosis of AD can delay the disease development and improve therapeutic effects, therefore, a diagnosis study of AD is urgent.

With the rapid development of neuroimaging technology, MRI technology has provided powerful support in AD research and has become an indispensable tool (Teipel et al., 2015). Since a significant improvement in the level of modern medical technology, it has been found that the causes of AD may involve many aspects in clinical research, including the brain region and gene abnormities (Heneka et al., 2015; Zhang et al., 2015; Olsson et al., 2016). As a result, the multimodal data fusion research of this disease is gradually becoming an emerging field, attracting widespread attention from researchers. At present, multimodal research can explore multiple potential pathogeneses of brain diseases and provided full details on the complementary advantages of information among various data points (Ning et al., 2018; Zhang et al., 2018b). For instance, Varol et al. (2017) presented the heterogeneity through discriminative analysis (HYDRA) method to classify diseased and healthy subjects through neuroimaging and genetic data of brain diseases. The results demonstrated that the two groups of subjects could be distinguished accurately. As the diverse modal data of each disease possesses different characteristics, it is a key point in exploring the problem of multi-factor pathogenesis of brain diseases to design a reasonable scheme according to these characteristics, which is also a hot topic worthy of sustained attention by researchers.

Moreover, owing to the various reasons, there are a few credible public databases for Alzheimer’s disease with multiple modal data such as neuroimaging, genes, proteins, and others, resulting in a small amount of available data (Zhang et al., 2018c). In addition, the data dimension is much higher than the unimodal in multimodal research (Zhang et al., 2018a; Peng et al., 2019; Zhang et al., 2019). These issues cause traditional methods to bottleneck with regard to data processing and analysis. However, machine learning methods have a strong adaptability in the processing of small sample data and high-dimensional data, which can already be maturely applied in many studies (Dai and Xu, 2013; Jiang et al., 2018; Komiske et al., 2018). For instance, Zhang et al. (2015) put forward a computer-aided diagnosis system based on machine learning for the classification of AD, reaching an average accuracy of 92.36%. Sun et al. (2018) proposed a novel machine learning based on the support of a vector machine to classify AD and NC, with an accuracy of 89.3%. These studies showed that machine learning achieved significant results in the field of discriminant analyses in brain science research, but those still include unilateral research such as data fusion, classification or feature extraction. Accordingly, the focus of this paper is on how to combine multimodal data of AD to design a complete brain science data analysis framework of data fusion, classification, feature extraction, and searching for lesions and disease-causing genes for the early diagnosis of this disease.

To better address the above problems, this paper will study multimodal data of neuroimaging and gene data of Alzheimer’s disease, and design a complete framework to realize classification, feature extraction and disease-causing factor extraction. Specifically, we first designed the fusion scheme of neuroimaging and genetic data to construct fusion features, then proposed a multimodal random forest (MRF) method to distinguish AD from NC to extract optimal fusion features. We further extracted abnormal brain regions and genes based on the optimal features. Compared to other methods, our framework was able to extract fusion features with higher differentiation ability and obtained higher classification accuracy, providing good insight into the causes of Alzheimer’s disease and a new solution for the early diagnosis of this disease. The Materials and Methods describes the experimental data and the framework for data fusion, classification, feature extraction, and pathogenic factor extraction. The Results shows the results of the experiment and performance comparison. The Discussion discusses the experimental results and the Conclusion summarizes this study.

## Materials and Methods

### Participants

In this paper, we collected a total of 72 participants from the ADNI database (http://adni.loni.usc.edu/) to appraise our presented framework, including 37 AD and 35 NC. The ADNI contains a variety of fMRI, structural MRI (sMRI), genetic data, etc. We selected the fMRI and genetic data of AD and NC, respectively, to implement our study. **Table 1** exhibits the baseline characteristics of participants and the statistic tests demonstrated no significant differences for the gender and age of patients and NC to guarantee no disturbances by other factors in the experiment (both *p* > 0.05). This research was supported by multiple governmental organizations and each participant provided written informed consent.

**Table 1:** Baseline characteristics of AD and CN.

Variables (Mean±SD)	AD(n =37)	CN(n = 35)	Statistic
Gender(M/F)	19/18	13/22	*p* = 0.324*
Age(years)	75.35 ± 7.949	77.14 ± 6.175	*p* = 0.291**

*The p value was gained by the chi-square test.

**The p value was gained by the two-sample t-test.

### Data Acquisition and Preprocessing

The fMRI data and corresponding genetic data were included in the experiment; therefore, we briefly describe the processes of the acquisition and preprocessing.

For fMRI data, all participants went through fMRI using a Philips Medical Systems 3T scanner at flip angle = 80.0 degree, 64.0×64.0 acquisition matrix, slices = 6720.0, slice thickness = 3.3 mm, TE = 30.0 ms and TR = 3000.0 ms. In order to facilitate the follow-up experiments, we carried out the preprocessing utilizing the DPARSFA software (Chao Gan and Yu Feng, 2010) based on the MATLAB platform. Concretely, the whole procedure is as follows: (1) file format conversion for the subsequent preprocessing; (2) the first 10 volumes are removed to eliminate instrument interference at the beginning of the scanning; (3) slice timing correction to make the time points between the two layers correspond; (4) head movement correction to exclude the effect of the movement of the participant’s head during the actual measurement; (5) normalization using the EPI template to eliminate individual differences of the participants; (6) smoothing to improve the effect of normalization and the signal-to-noise ratio; (7) detrending to eliminate the impact of Volex’s signal fluctuations on the results; (8) filtering 0.01-0.1HZ to retain low frequency signals.

For genetic data, we collected the data on the Ilumina Omni 2.5M BeadChip from ADNI and used the PLINK software to perform the quality control. The PLINK is an open-source, free toolset used for the genotype/phenotype data analysis. Briefly, the procedure included sample call rate, genotyping, minimum allele frequency and the Hardy-Weinberg equilibrium test, the values of which were set to 95%, 99.9%, 4%, and 1E-4, respectively.

### Multimodal Fusion Analysis Framework

**Figure 1:**
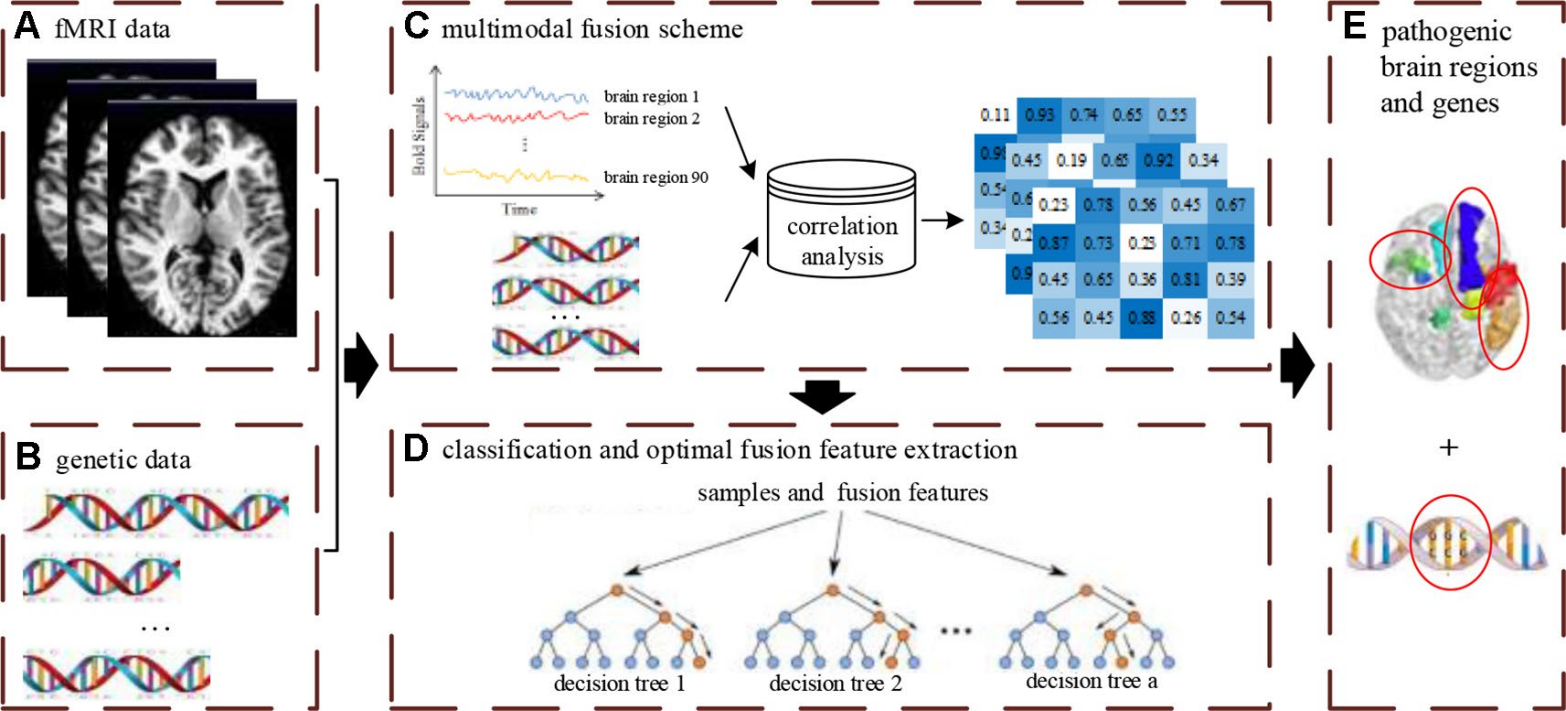
The overview of multimodal fusion analysis framework. The **(A)** denotes unprocessed fMRI data. The **(B)** denotes unprocessed gene data. The **(C)** denotes the fusion process of multimodal data. The **(D)** denotes the construction process of multimodal random forest model for classification and optimal feature extraction. The **(E)** denotes the extraction results of pathogenic brain regions and genes by feature fusion scheme and multimodal random forest model.

The multimodal data fusion analysis was carried out using a novelty framework to effectively supply accurate classification and discriminative optimal fusion features which were further analyzed to locate the pathogenic brain regions and genes. The integrated analysis framework is shown in **Figure 1** and includes three main parts: (1) designing of the feature fusion scheme of neuroimaging and genetic data; (2) carrying out classification and optimal fusion feature extraction using the MRF method; (3) locating the pathogenic brain regions and genes, where the **Figure 1A** denotes the input data of the framework.

#### Fusion Feature Construction

Designing a reasonable data fusion scheme is a prerequisite of constructing features to realize a multimodal fusion analysis framework. Specifically, for the genetic data of each participant, it is stored in the form of the single nucleotide polymorphism (SNP) in a chip. In order to extract genetic data, we need to further process the SNPs that have been done for quality control. First, these SNPs are grouped in genes and the gene groups with SNPs above the threshold are kept to ensure experimental accuracy. To integrate with fMRI data, we segment the SNPs in each gene, which keeps the length of all gene sequences consistent. Then these gene sequences are digitally encoded, converting A to 1, T to 2, C to 3, and G to 4. For the fMRI data of each participant, we divide the brain image obtained by pre-processing into 90 brain regions using the AAL template. To match the genetic data, we cut the length of the time series for each brain region to equal the length of the gene sequence. After the two types of data are both processed, we use the Pearson correlation analysis to estimate the correlation between each brain region and each gene and regard the calculated correlation coefficients as fusion features. The data fusion method of each participant is the same as above, and we eventually obtain the fusion features of all participants.

#### Multimodal Random Forest Construction

The multimodal random forest model is the core of the multimodal fusion analysis framework, which is aimed at analyzing multimodal data. Suppose there are *N* samples and *M* fusion features in this study, then all samples are represented as {(*s*_1_,*y*_1_), (*s*_2_,*y*_2_), ,(*s* *_n_*,*y* *_n_*)}, where *y* *_n_* is the classification label. The *N* samples and *M* fusion features are seen as the input of the MRF. In order to better train the MRF model, the sample set is divided into a training set and a testing set according to the partition ratio of 6:4, before constructing the MRF model. The detailed construction procedure is shown below. Initiatively, *n* samples and *m* fusion features are randomly selected to form a decision tree which is constructed using the classification and the regression tree (CART) algorithm. Subsequently, we set the number of decision trees in the MRF model to *a*. Finally, *a* decision trees are integrated to complete the construction of MRF.

Since the performance of the MRF model underlying different number of decision trees may be discrepant, we set the number to different values and selected the most suitable number to build the MRF model, ensuring optimal performance.

#### Classification and Feature Extraction

Classification and feature extraction are two important applications of the MRF model. In this study, the MRF model is adopted for AD classification to validate the effect of multimodal data fusion. Because there are two groups of samples including AD and CN, we set the labels to “–1” and “+1”. The optimal MRF model is constructed by the above methods. We used the majority voting method to predict the sample label of the test set. When the test sample is used to assess the model, each base classifier will give a classification result. If the results of most base classifiers are “–1”, the prediction label is AD, otherwise, it is CN.

When the optimal MRF model is built, the test set is reclassified to achieve the highest value of the classification accuracy. We count the *m* fusion features in each base classifier and sort the frequency of each fusion feature in descending order to select the first *b* fusion features for further analysis. The *b* fusion features are divided into several subsets with the number of features increased in turn, and the discrimination ability of each subset is evaluated by the MRF model. Eventually, the most discriminatory fusion feature subset is selected as the optimal fusion feature subset.

#### Pathogenic Brain Regions and Genes Extraction

Identifying abnormal brain regions and disease-causing genes is the ultimate goal of designing the multimodal data fusion analysis framework. The chosen optimal fusion features include two parts of the brain region and genes. More importantly, we extracted and analyzed the two parts separately. We counted the frequencies of the corresponding brain regions in the optimal fusion features. The higher the frequency is, the more influential the brain region is on the disease. Similarly, we also calculated the frequencies of genes. The higher the frequency is, the more strongly the gene is associated with the disease.

### Performance Evaluation

With the aim of evaluating the performance of the MRF model, the classification accuracy is used as an evaluation index to determine whether the model can carry out accurate classification for multimodal data, as shown in formula (1).

(1) $$C=\frac{\Sigma_{i=1}^{k}S_{i}}{T}$$

where *C* represents the accuracy of MRF and *T* represents the total of the testing set. When the *i^th^* participant is predicted precisely, the value of *s_i_* is 1, otherwise, it is 0.

Additionally, we further employed other conventional correlation analysis methods such as canonical correlation analysis (CCA) and correlation distance (CD) to calculate the correlation between the brain region and genes and combined these correlation analyses with other classification or feature extraction methods to form several frameworks. These frameworks are compared to the proposed multimodal fusion analysis framework and the comparison results are shown in part 4 of Section 3.

## Results

### Constructing Fusion Features

After data preprocessing, fMRI images of each sample were segmented into 90 brain regions, and 82400 SNPs were preserved in the genetic data. We grouped the remaining SNPs according to their corresponding gene and selected 36 groups with SNPs counts of more than 30. Next, the Pearson correlation coefficients between the 36 gene groups and 90 brain regions were calculated according to the feature construction approach mentioned in the *Methods and Materials* section, and the fusion feature matrix of 36×90 was formed. These fusion features were the initial input features used to construct the multimodal random forest.

### Training the Optimal Multimodal Random Forest

**Figure 2:**
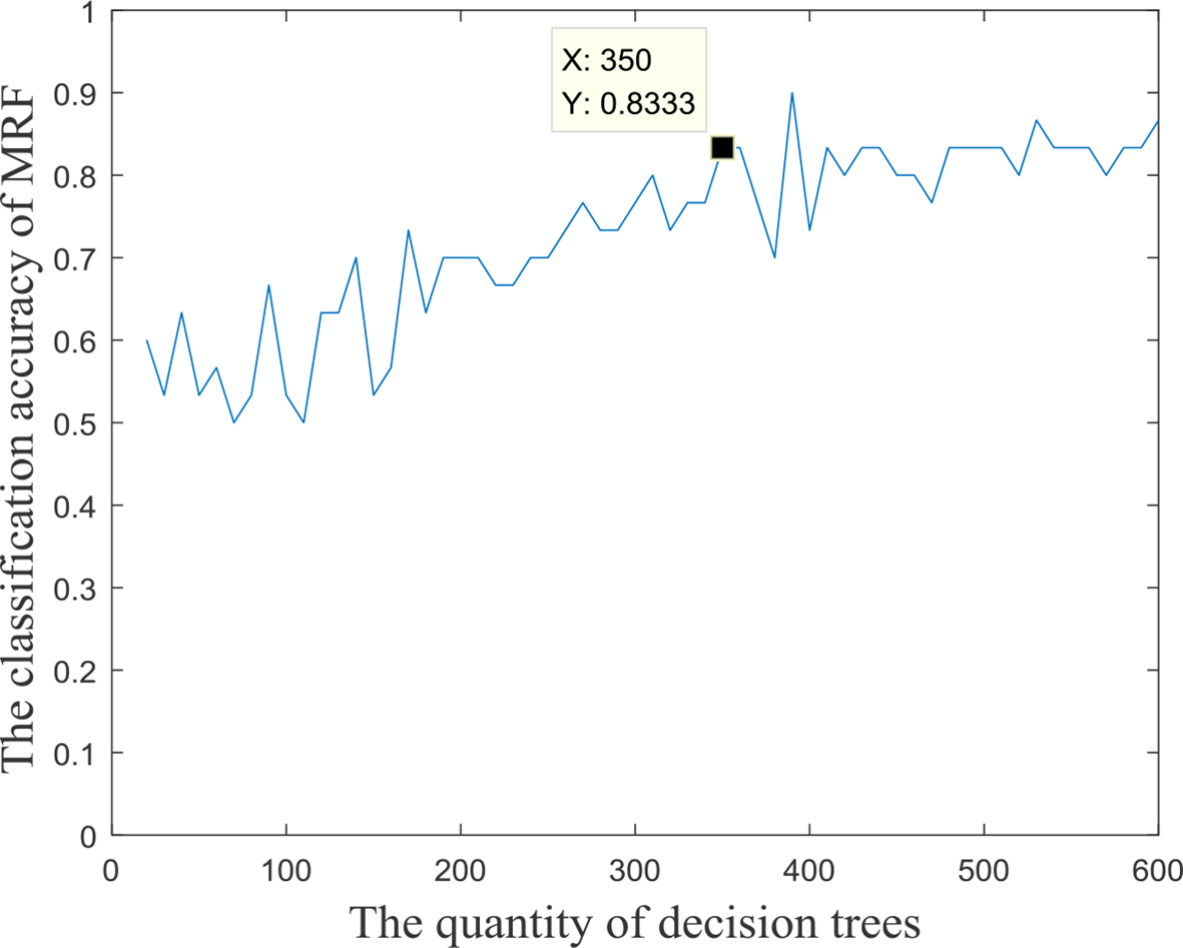
The accuracy of MRF with different quantities of base classifiers.

The input features’ number of each decision tree in the random forest was 57, and these input features were randomly extracted from all the 3,240 fusion features. In the experiment, we found that the quantity of decision trees had a significant impact on the performance of the multimodal random forest. If the number of decision trees is too small, it is difficult to reflect the advantages of ensemble learning. If the number is too large, it will lead to greater similarity and redundancy between decision trees, which will also decrease the accuracy of the ensemble learner. Specifically, by testing different numbers of decision trees, the search interval of the optimal number was initially determined [0,600]. We then took 10 as the search step, and the performances of multimodal random forests with different numbers of decision trees were evaluated. The result is exhibited in **Figure 2**. We can learn from the figure, that after the quantity of decision trees reached 350, the performance of the multimodal random forest tended to be stable, and its stability value was about 83.3%. In addition, although there were several points in the curve whose classification performance was higher than the stable value, these points were caused by the random fluctuations of performance, which were not stable and cannot be used as the optimal number of decision trees. Therefore, a value of 350 was considered to be the optimal number of decision trees.

### Extracting Fusion Features With the Strongest Recognition Abilities

It is widely accepted that high classification accuracy is closely related to the validity of input features. A decision tree with high accuracy in a multimodal random forest provides a reference for extracting the most recognizable fusion features. More precisely, the top 100 decision trees with the best classification performance in the optimal multimodal random forest were retained, and the input features of these decision trees were counted. Next, with frequency as the criterion, we retained 400 high-frequency input features. Therefore, the search range of the most recognizable fusion feature was reduced from 3240 to 400 dimensions.

**Figure 3:**
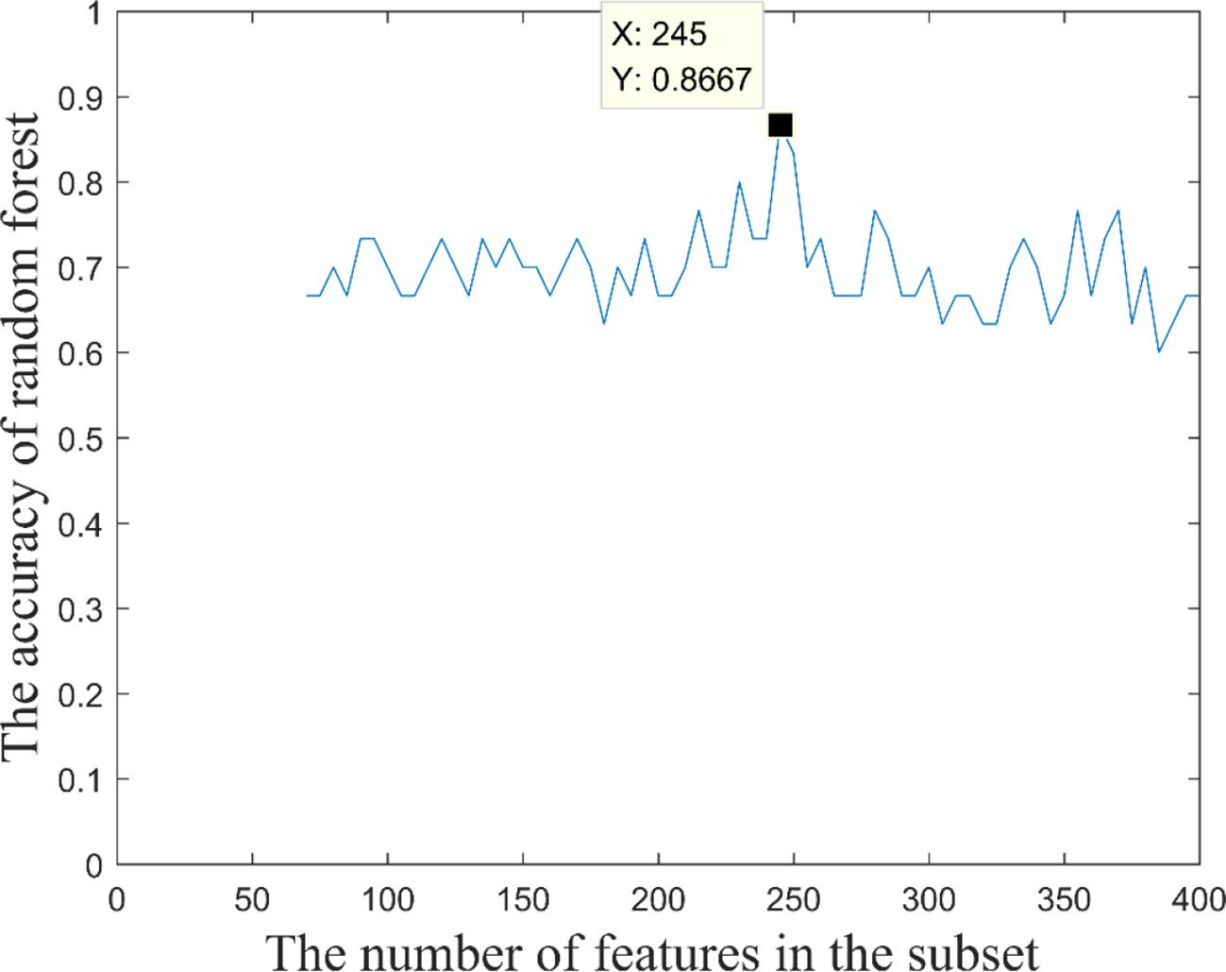
Classification performance of feature subset with different numbers of features.

**Figure 4:**
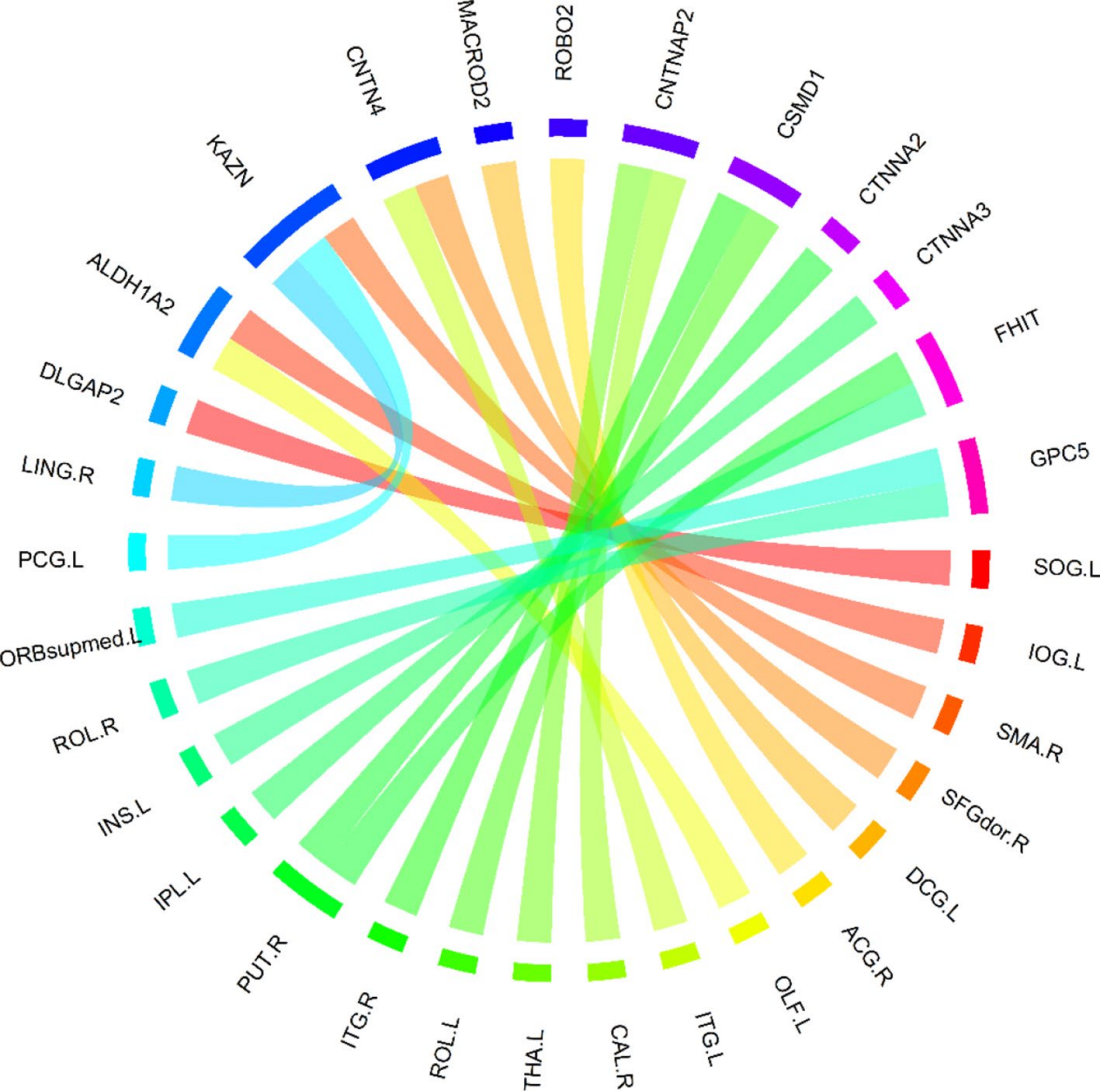
The top 20 features with the strongest recognition abilities.

Based on these 400 high-frequency features, we further searched the most effective feature subset. Initially, 400 high-frequency features were divided into 67 subsets according to frequency. The number of features in the subset increased gradually with the step size of 5, and the minimum number and the maximum number of features in subset were 70 and 400. Subsequently, we constructed a multimodal random forest to test the classification performances of different feature subsets using the methods mentioned above, and the results are shown in **Figure 3**. As shown in the figure, when the number of features in the subset exceeded 245, the trend of classification performance curve changed from rising to falling. The change suggests that if the number of optimal features is less than 245, some important features may be neglected. Otherwise, some redundant or invalid features may be included in the analysis. Therefore, the first 245 fusion features were considered as fusion features with the strongest recognition abilities, and the first 20 features of those are shown in **Figure 4**. The nodes in the figure represent brain regions or genes, and the edges indicate the associations between brain regions and genes.

### Comparison of Classification Performance

To verify the validity of the most recognizable fusion features, the multimodal random forest was compared with several other typical methods, and the specific results are shown in **Table 2**. We found that there were significant differences in the number of the most recognizable fusion features extracted by different methods. The method of combining Pearson with the random SVM cluster (Pearson + RSVMC) had the largest number of features, while our method had the least number of features. More interestingly, the most recognizable features extracted by our method had the best classification performance. The fact indicated that although the number of feature subsets extracted by other methods was larger, they still contained redundant or invalid features. In addition, we compared the overlaps between other methods and our method in extracting the most recognizable feature subset and found that the size of the overlap was positively correlated with the performances of other methods. The hypergeometric test further proved that these overlaps were not randomly generated, which also confirmed the effectiveness of our method in extracting the most recognizable fusion features.

**Table 2:** The performance comparison of different methods.

Method	Discoveries	Classification accuracy of SVM	Overlap with our method
Pearson + MRF	245	0.8667	—
Pearson + RSVMC	400	0.8000	135 (p = 5.710129e-23)
Pearson + t-test	351	0.7000	88 (p=1.054523e-11)
CCA + t-test	313	0.7667	116 (p=1.883298e-06)
DCA + t-test	329	0.7333	99 (p=4.343267e-14)

**Figure 5:**
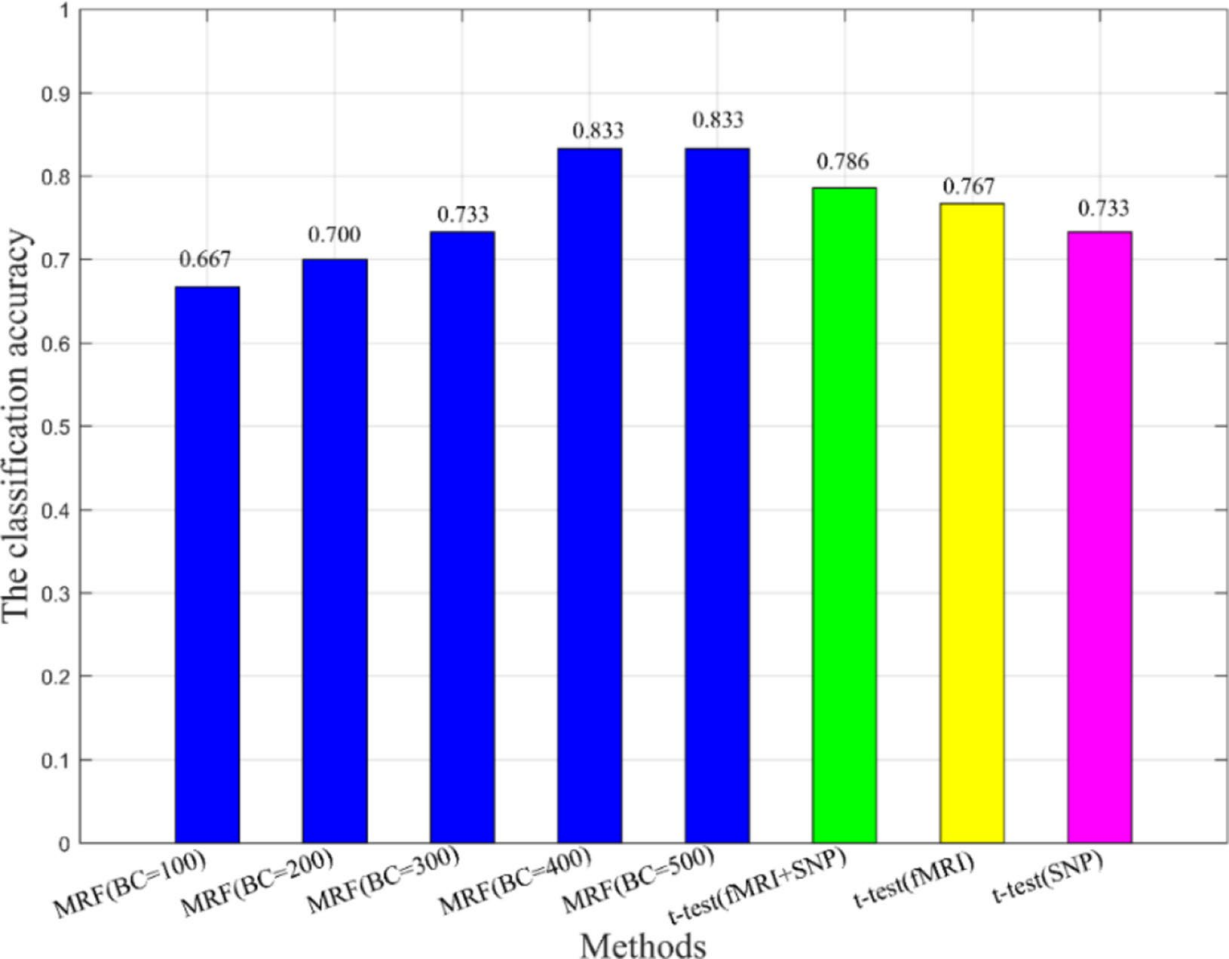
The comparison of multimodal method and unimodal method.

Additionally, we also compared the multimodal method with the unimodal method to verify the effectiveness of fusion features in classification (see **Figure 5**). In **Figure 5**, BC is the number of base classifiers, the classification accuracies of all MRFs are obtained by multimodal experiments. The t-test means that features are extracted by t-test and the SVM is used as the classifier. When the classical two-sample t-test method was used for classification, the classification accuracy under the multimodal condition was higher than that under the unimodal condition, which indicates that the multimodal method has better performance advantages. On the other hand, we found that the machine learning method used in this paper, after training and optimization, might have more advantages than the conventional statistical methods under the condition of multimodal data.

### Analyzing Abnormal Brain Regions and Genes

**Figure 6:**
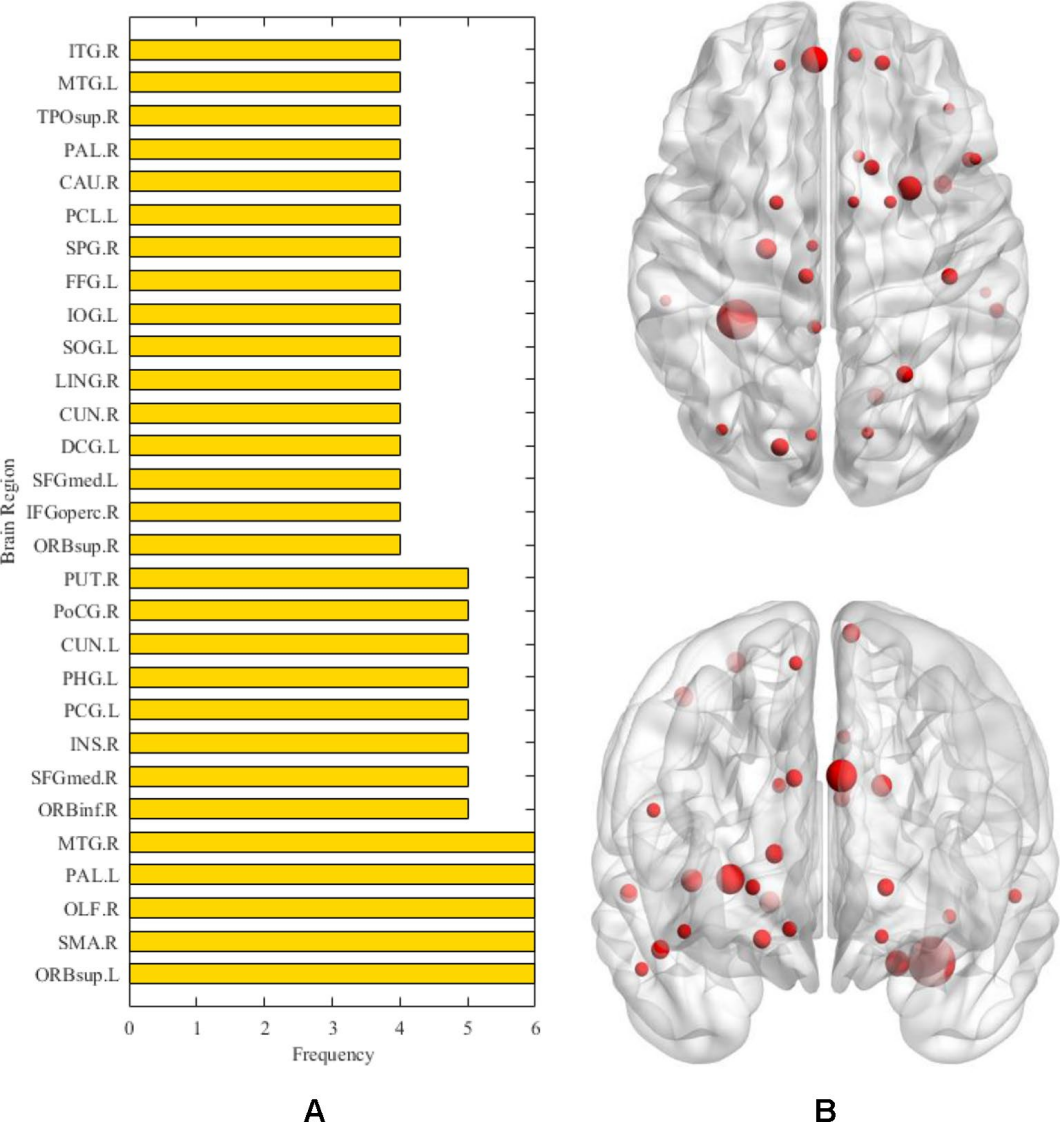
The **(A)** denotes the frequencies of abnormal brain regions related to AD. The **(B)** denotes the location of the corresponding abnormal brain regions.

**Figure 7:**
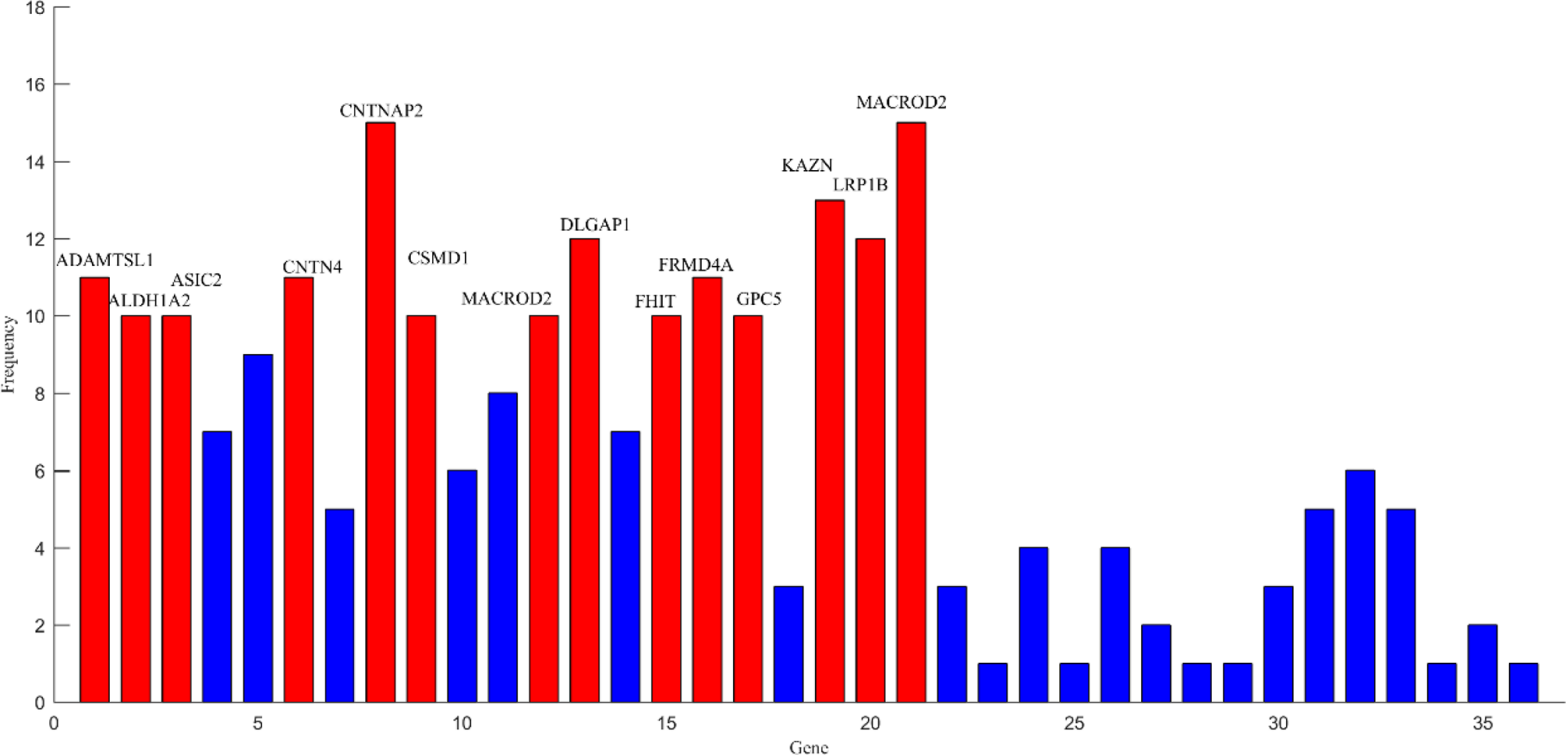
The frequencies of main pathogenic genes.

After the above comparative experiments, we were confident that the most recognizable feature set extracted by our method was more reasonable. We split these fusion features and isolated specific brain regions and genes. If the brain region or gene appeared repeatedly in the most recognizable feature set, it means that the brain region or gene is closely related to AD. The abnormal brain regions and genes found in this study are shown in **Figure 6** and **Figure 7**, respectively. The abnormal brain areas include the olfactory cortex, insula, posterior cingulate gyrus, and the lingual gyrus. The abnormal risk genes include CNTNAP2, LRP1B, FRMD4A and DAB1.

## Discussion

### Methodological Considerations

Exploring the etiologies of AD is a long-term challenge in brain science. Our work provides a scalable framework for this field. Unlike most previous studies on multimodal data fusion, most of them are limited to neuroimaging data (Palesi et al., 2016; Whitwell, 2018), such as fMRI-EEG fusion (Brueggen et al., 2017). In this paper, gene and fMRI data are fused and analyzed using machine learning approach. The results show that there are some correlations between brain regions and genes, and these correlations can provide references for the detection of abnormal brain regions and potential risk genes of AD.

In this study, a framework for multimodal data fusion analysis was constructed. As an important part of the framework, we evaluated various correlation analysis methods, including CCA and CD (Lei et al., 2016; Ponsoda et al., 2017). The experimental results show that compared with other more complex methods, the Pearson correlation analysis is more applicable and explanatory in fusion analysis owing to its unique advantages in detecting direct linear correlation (Yang et al., 2018). In current research, the direct correlations between brain regions and genes are more helpful in explaining the interactions of genes and the brain structure in the pathological mechanism of AD (Grabert et al., 2016).

On the other hand, the multimodal random forest, based on fusion features, has more obvious advantages than the traditional single-modal method (Chanel et al., 2016; Guo et al., 2017). For instance, Rosa et al. (2015) used sparse network-based models to identify brain disease patients with an accuracy rate of 79%, and Li et al. (2018) employed group-constrained sparse inverse covariance which achieved about 80% accuracy in AD recognition. The average classification accuracy of the multimodal random forest is 83.33%. One of the reasons is that our method relies on information complementarity between different modal data. Another reason is that the internal parameters of the multimodal random forest are optimized, such as the number of input parameters in a decision tree and the number of decision trees in a random forest, which makes the ensemble of base classifiers more efficient.

Moreover, all participants are partitioned into the training set and the test set according to the partition ratio of 6:4. The training set is utilized to construct the multimodal random forest. The test set is utilized to measure the classification performance of the multimodal random forest. In the process of the MRF construction and measurement, the samples and features are randomly selected, which avoids over-optimization to a certain extent. In addition, the experimental results reveal that the multimodal random forest accomplishes well on the real dataset, therefore, there is no possibility of an over-optimized model.

### Abnormal Brain Regions and Genes

In this study, we extract brain regions and genes related to AD based on the most discriminative fusion features. These factors are validated from various perspectives in the following paragraphs.

First, in the detection of abnormal brain areas, we found that the frequencies of some brain areas were significantly higher, such as olfactory cortex, insula, posterior cingulate gyrus and lingual gyrus, which meant that these brain areas played an important role in the progression of AD. Specifically, the olfactory cortex participates in the encoding of episodic memory in the brain (Gottfried et al., 2004).The olfactory cortex of AD patients shows obvious neurodegeneration, the number of neurofibrillary tangles and neuropathic plaques increase (Reyes et al., 1993), whereas the volume and activity intensity decreases significantly (Vasavada et al., 2015), which leads to memory impairment in AD patients, especially the decline of olfactory memory. The insula is also an abnormal brain region detected in this study. Previous studies have found that the pattern of emotional contagion in AD patients is different from that in normal people, showing a primitive form of empathy, which is closely related to the insula (Choi and Jeong, 2017). On the other hand, morphological abnormalities are detected in the insula of AD patients (Zhang et al., 2015; Petrides et al., 2017). This brain area of AD patients shows obvious atrophy (Trzepacz et al., 2013), and with the thinning of cortical thickness, the cognitive decline of AD patients becomes increasingly severe (Möller et al., 2016). It is worth noting that the abnormal brain areas detected in this study include the posterior cingulate gyrus, which has been reported many times as a typical brain area seriously affected by AD. In general, the posterior cingulate cortex is the area of scene construction (Irish et al., 2015), while posterior cingulate gyrus synaptic function in AD patients is affected in the precursor stage of the disease, and may become the basis of some early clinical sequelae related to AD (Scheff et al., 2015). More evidence suggests that functional connectivity, regional cerebral blood flow, and glucose metabolism of the posterior cingulate gyrus are also abnormal in AD (Iizuka and Kameyama, 2017; Scheltens et al., 2018; Yamashita et al., 2019). Besides the typical brain areas mentioned above, more abnormal brain areas with a subtle association to AD are also detected, including the lingual gyrus and fusiform gyrus. In recent studies, irregularities of β-amyloid loaded in the fusiform gyrus underline the abnormal facial recognition mechanism of AD patients (Ishiki et al., 2015; Chang et al., 2016), and the neurodegeneration in the lingual gyrus may be associated with atypical cognitive variations in AD patients (Phillips et al., 2018). The detection of these brain regions proves the validity of the methods used in this paper, and helps to explain the cooperation of many brain regions in the pathogenesis of AD.

The detection and analysis of AD risk genes are significant contributions of our paper. We found that some potential risk genes for AD included CNTNAP2, LRP1B, FRMD4A, and DAB1. The polymorphism in the CNTNAP2 gene has been found to take part in many aging diseases (Iakoubov et al., 2015). The result of a genome-wide association analysis indicates that this gene is a novel susceptibility loci of AD (Hirano et al., 2015). More precisely, the direct downregulation of the CNTNAP2 gene in the hippocampus and other regions may be the key pathogeny of AD (van Abel et al., 2012). LRB1B is another high-frequency gene detected by our study. Previous research has shown that haplotypes in the LRP1B gene can protect the aged from cognitive decline (Poduslo et al., 2010). Silencing of the LRP1B gene expression in AD patients may induce abnormal responses of complementary proteins (Benoit et al., 2013). Additionally, we also observed that the FRMD4A gene may be a risk gene for AD. Lambert et al. (2012) identifies FRMD4A as a new genetic risk factor for AD through a genome-wide haplotype association study. Further studies suggest that FRMD4A may play a pivotal part in amyloid protein formation and tau-related pathways in AD (Martiskainen et al., 2015). The association between the DAB1 gene and AD is also noticed in our study, which is consistent with the result of functional enrichment analysis (Gao et al., 2015). In fact, a recent study also shows that the expression of the DAB1 gene in the cerebral cortex is up-regulated, which leads to abnormal synthesis of many proteins in the brains of AD patients (Muller et al., 2011). The discovery of these risk genes will assist in understanding the pathogenesis of AD from a genetic perspective.

### Limitations and Future Directions

This study has made some progress in the multimodal fusion of brain science, but some limitations should be mentioned. We proposed the MRF model to classify AD and CN using the correlations between brain regions and genes and achieved satisfactory results. However, because the Pearson correlation analysis still possibly neglects subtle correlations, we will design a more appropriate correlation analysis method to construct fusion features in a follow-up study. On the other hand, although we detected the most discriminative fusion features, and proved that there is a certain correlation between brain region and gene, we still want to determine the specific pathways of different genes affecting the brain regions, and therefore still need to continue to invest a lot of energy in this research.

## Conclusion

Unlike previous classical unimodal brain science research, this paper attempts to carry out multimodal fusion research on AD based on fMRI and gene data. Our work first validates the potential of the associations between brain regions and genes in the accurate recognition of AD and proposes the fusion features of brain regions and genes. We then constructed a multimodal random forest according to the fusion features. The multimodal random forest and feature construction method are integrated into a comprehensive framework based on machine learning. With this framework, we have realized the efficient detection of AD patients, and located the pathological brain regions and potential risk genes of AD. Our research can provide references for precision medicine in AD.

## Data Availability Statement

All data files are available from the ADNI (http://adni.loni.usc.edu/) database with no accession number(s).

## Ethics Statement

This study was carried out in accordance with the recommendations of National Institute of Aging- Alzheimer’s Association (NIA-AA) workgroup guidelines, Institutional Review Board (IRB). The study was approved by IRB of each participating site, including the Banner Alzheimer’s Institute, and was conducted in accordance with Federal Regulations, the Internal Conference on Harmonization (ICH), and Good Clinical Practices (GCP). Written informed consent was obtained from the individual(s), and minor(s)’ legal guardian/next of kin, for the publication of any potentially identifiable images or data included in this article.

## Author Contributions

X-AB proposed the design of the work and revised it critically for important intellectual content. YL carried out the experiment for the work and drafted part of the work. RC and YW collected, interpreted the data and drafted part of the work. All the authors approved the final version to be published and agreed to be accountable for all aspects of the work in ensuring that questions related to the accuracy or integrity of any part of the work are appropriately investigated and resolved.

## Funding

This work was supported by the Hunan Provincial Science and Technology Project Foundation (2018TP1018), the National Science Foundation of China (No. 61502167).

## Conflict of Interest

The authors declare that the research was conducted in the absence of any commercial or financial relationships that could be construed as a potential conflict of interest.
